# Effects of inorganic or organic selenium on immunoglobulins in swine

**DOI:** 10.1186/2049-1891-4-47

**Published:** 2013-11-27

**Authors:** Ashley Gelderman, Jeffrey Clapper

**Affiliations:** 1 Department of Animal Science, South Dakota State University, Brookings 57007SD, USA

**Keywords:** Immunoglobulin, Pig, Selenium

## Abstract

A study was conducted to determine if Se source fed during gestation and lactation affects passive transfer of immunoglobulins. Sixty days prior to breeding (d -60), gilts were randomly assigned to one of three treatments prior to breeding and throughout gestation: control (Control, no supplemental Se; n = 8), inorganic Se (Inorganic Se, 0.3 ppm; n = 4) and organic Se (Organic Se, 0.3 ppm; n = 4). Blood was collected on d -60, 57 and 113 of gestation and on d 21 of lactation and milk was collected at d 0, 1, 7, 14, and 21 of lactation. Blood was collected from piglets at d 0, 1, 7, 14, and 21 of age. Gilts fed organic Se had greater (*P* < 0.05) circulating concentrations of Se than Inorganic and Control gilts. Regardless of treatment, circulating concentrations of Se were greatest *(P* < 0.05) at d -60 compared to all other days. Serum concentrations of IgG were greatest (*P* < 0.05) in gilts at d 57 of gestation compared to d 113. Serum concentrations of IgA were greatest (*P* < 0.05) on d 113 of gestation and d 21 of lactation compared to d -60 and 57. Serum concentrations of IgM were greater (*P* < 0.05) at d 57 compared to d -60. Inorganic gilts had greater (*P* < 0.05) colostral and milk concentrations of IgG and IgM than Organic or Control gilts. Circulating concentrations of Se in piglets were greatest (*P* < 0.05) at d 14 and 21 of age compared to all other days. Piglets from gilts supplemented with organic Se had greater (*P* < 0.05) circulating concentrations of Se on d 1 versus piglets from gilts supplemented with no additional Se. The immunoglobulin concentrations of IgG, IgA, and IgM were lowest (*P* < 0.05) on d 0 and then increased when compared to d 1. The addition of different sources of Se did not affect the immunoglobulin concentrations in the gilt or piglet.

## Background

Traditionally, Se is known to be toxic or harmful to animals in certain regions in the U.S., but Se deficiency is a more widespread problem. Selenium was not considered important until Eggert et al. [1] reported that Se is an essential nutrient in swine because it helped prevent hepatosis diaetetica. One of the main biochemical functions of Se is as a component of glutathione peroxidase [2]. Typically, Se is provided as a supplement in swine diets, either in the inorganic (sodium selenite) or organic (enriched Se yeast) forms. The current recommendation for the inclusion of Se in diets is 0.30 ppm [3].

Feeding organic Se compared to inorganic Se resulted in greater serum concentrations of Se in the females as well as progeny [4] and greater colostral concentrations of Se [5]. When feeding either inorganic or organic Se forms, there were no differences in total pigs born, litter average daily gain (ADG), or litter weight [6]. Furthermore, the addition of either inorganic or organic Se at 0.3 ppm reduced the number of stillborn piglets [7]. At birth, piglets had greater whole blood concentrations of Se when born to sows fed organic Se than piglets born to sows fed the control diet [7].

Colostrum and milk are essential for the survival of piglets as sources of digestible nutrients [8] and helps to establish passive immunity [9,10]. Increased IgG absorption and enhanced humoral immune function has been shown in diets with supplemental Se [11,12]. Research regarding the effects of adding Se to maternal diets to increase serum concentrations of IgG is equivocal [13-15]. Sows injected with Se had increased colostral concentrations of IgM compared to other treatment groups, indicating a basis for enhanced passive transfer of antibodies [16]. Beef cows supplemented with Se have also been shown to have increased colostral concentrations of IgG [17]. Thus, Se supplementation may prove useful in enhancing the immune profile in the newborn pig. Therefore, the objective of the current study was to determine if diets supplemented with an organic or inorganic source of Se would increase whole blood Se and serum concentrations of IgG, IgA, and IgM in gilts and their progeny.

## Methods

### Experimental design

Twenty-seven crossbred gilts (Yorkshire × Landrace × Hampshire; 130 ± 6.44 kg; approximately 240 d of age) were used in this experiment. Gilts were fed 1.81 kg/d of their respective diet beginning 60 d prior to breeding. Gilts were randomly assigned to one of the three dietary treatments: control (Control) diet with no supplemental selenium added (n = 9), an inorganic source of Se (Inorganic) with 0.3 ppm of Se added as sodium selenite (n = 9), and an organic source of Se (Organic) with 0.3 ppm of Se added as selenized yeast (n = 9). Diets were formulated to meet the 1998 NRC requirements for gestating swine (Table 1). Concentrations of Se in the corn and soybean meal used in the gestation and lactation diets were 0.02 ppm and 0.759 ppm, respectively. Diets contained 0.182 ppm, 0.469 ppm, and 0.419 ppm of Se for the control, inorganic source, and organic source diets, respectively. Gilts were individually housed in a 1.2 m × 1.8 m partially slatted pen in an environmentally controlled room with ad libitum access to water. Each gilt was fed 15 mg altrenogest (Matrix) each day for 15 d to synchronize estrus. Estrus detection was performed by exposing gilts to a mature boar twice daily for 20 min each, beginning the third day after cessation of the altrenogest treatment and continuing for 4 d. The first day the gilt stood immobile in the presence of the boar was designated as d 1 of the estrous cycle. Gilts were bred by artificial insemination 2 h after their initial display of standing estrus. Pregnancy was confirmed by ultrasound 28 d post breeding. Of the 27 females bred, 16 gilts became pregnant; Control (n = 8), Inorganic (n = 4), and Organic (n = 4).

**Table 1:** Maternal diets formulated to meet or exceed 1998 NRC recommendations for lactating sows

	**Lactation diets**		
**Ingredient, %**	**No Se**	**Inorganic Se**	**Organic Se**
Corn	65.15	65.15	65.15
Soybean meal, 44%	27.50	27.50	27.50
Soybean oil	4.00	4.00	4.00
Dical, dynafos	1.60	1.60	1.60
Limestone	0.90	0.90	0.90
Salt	0.40	0.40	0.40
Vitamin premix^3^	0.30	0.30	0.30
Trace mineral	0.15^1^	0.15^2^	0.15^1^
Se^4^	-	-	0.05

^1^Trace mineral mix containing no sodium selenite.

^2^Trace mineral mix containing at least 200 ppm Se as sodium selenite.

^3^Vitamin premix contained the following quantities of vitamins and microminerals 4,545,455 IU vitamin A/kg, 681,818 IU vitamin D_3_/kg, 39,182 IU vitamin E/kg, 1,818 mg vitamin B12/kg, 1,818 mg menadione/kg, 164 mg biotin/kg, 6,250 mg pyridoxine/kg, 4,091 mg riboflavin/kg, 1,364 mg thiamine/kg, 110,000 mg Zn/kg, 110,000 mg Fe/kg, 29,000 mg Mn/kg, 11,000 mg Cu/kg, 240 mg I/kg, 200 mg Se/kg.

^4^ Sel-Plex® premix: Total selenium 600 ppm.

Gilts were bled via jugular venipunture 60 d prior to breeding, d -60, (2 h after eating), d 57 and 113 of gestation, and d 21 of lactation. Blood was separated into two aliquots of approximately 2.5 mL each to be used for analysis of whole blood concentrations of Se and serum concentrations of immunoglobulins. Serum was collected by allowing blood samples to clot overnight at 4°C before centrifugation (1,500 × *g* for 30 min) and storage at -20°C. At 5 and 2 wk pre-farrowing, gilts were administered a 2 mL dose of Rhinogen BPE (Intervet, Inc., Millsboro, DE) intramuscularly. Gilts were transferred to their farrowing pens on d 107 of gestation where they had ad libitum access to water and fed 1.81 kg/d of their respective diets. Parturition was induced by administering 10 mg PGF_2α_, i.m. (Pfizer; New York, NY) on d 113 to ensure attendance at the start of farrowing. During lactation, diets (Table 2) were offered ad libitum and formulated to meet or exceed 1998 NRC recommendations for lactating sows. The concentration of Se in the lactation diets were 0.272 ppm, 0.523 ppm, and 0.547 ppm for the control, inorganic source, and organic source, respectively. Sows were milked at farrowing after birth of the first piglet (d 0) and on d 1, 7, 14, and 21 of lactation for determination of colostral and milk concentrations of Se and immunoglobulins. Prior to milking on d 1, 7, 14, and 21, piglets were removed for at least 1 h and dams were administered 10 mg of oxytocin i.m. (VetTech; Shippack, PA) to facilitate milk let down. Colostrum and milk samples were collected and immediately frozen at -20°C for subsequent analysis of Se and immunoglobulin concentrations.

**Table 2:** Serum concentrations of IgG, IgA, IgM in gilts fed no additional Se supplementation (control), inorganic source of Se supplementation, or an organic source of Se supplementation

	**Treatment groups**			
**Item**	**Control (n = 8)**	**Inorganic Se (n = 4)**	**Organic Se (n = 4)**	***P*-value**
IgG, mg/mL	8.56 ± 1.16	9.48 ± 1.64	9.78 ± 1.65	0.805
IgA, mg/mL	0.25 ± 0.09	0.20 ± 0.13	0.37 ± 0.13	0.627
IgM, mg/mL	3.16 ± 0.42	3.04 ± 0.59	3.29 ± 0.59	0.958

Data are expressed as least square means ± SEM.

To ensure that no pig had suckled prior to sampling, piglets were removed from their dam, dried off, and placed in plastic totes that had a covering of wood chips at the bottom and a heat lamp located above the tote. Three piglets from each litter were randomly selected and bled via jugular venipuncture prior to suckling (d 0) and on d 1, 7, 14, and 21 for determination of whole blood Se and serum concentrations of immunoglobulin. Blood was separated into two aliquots to be used for analysis of whole blood concentrations of Se and serum concentrations of immunoglobulins. Serum was collected by allowing blood samples to clot overnight at 4°C before centrifugation (1,500 × *g* for 30 min) and storage at -20°C. Whole blood was frozen at -20°C for later analysis of circulating concentrations of immunoglobulins.

All procedures involving animals were approved by the Institutional Animal Care and Use Committee of South Dakota State University. All animals were housed and cared for in accordance with the Guide for the Care and Use of Animals in Agriculture Research (2010).

### Immunoglobulin analysis

Serum concentrations of IgG, IgA, and IgM were quantified in piglet and sow serum by ELISA (Bethyl Laboratories, IgA E101-102, IgG E101-104, and IgM E101-100, Montgomery, TX). The assay was conducted in 96-well, high binding microtiter plates (NUNC-Immuno Plate, 446612, VWR International Batavia, IL). The assay for each immunoglobulin was conducted according to manufacturer recommendations. Standards were prepared according to manufacturer’s instructions and pipetted into duplicate wells. Sow serum was diluted in sample/conjugate diluent (50 mmol/L Tris, 0.14 mol/L NaCl, 1% BSA, 0.05% Tween 20) to 1:120,000, 1:1,000, and 1:10,000 for IgG, IgA, and IgM, respectively. Sow colostrum and milk samples were diluted in sample/conjugate diluent to 1:250,000 (d 0), 1:100,000 (d 1), and 1:10,000 (d 7, 14, and 21) for IgG. Sow colostrum and milk samples were diluted to 1:100,000 (d 0), 1:25,000 (d 1), and 1:10,000 (d 7, 14, and 21) for analysis of IgA. Colostrum and milk samples for IgM were diluted to 1:25,000 (d 0) and 1:10,000 (d 1, 7, 14, and 21). Piglet serum was diluted to 1:40,000, 1:5,000, and 1:5,000 for IgG, IgA, and IgM, respectively.

Absorbance was read at 450 nm (Molecular Dynamics, Spectramax Plus 384). Intra assay CVs were 5.02%, 4.96%, and 3.94% for IgG, IgA, and IgM, respectively. The inter assay CVs were 13.70%, 16.18%, and 14.40% for IgG, IgA, and IgM, respectively. Sensitivity of the ELISA was 5.19 ng/mL, 12.01 ng/mL, and 12.23 ng/mL for IgG, IgA, and IgM, respectively.

### Milk and colostrum samples

After thawing, colostrum samples were centrifuged at 9,700 × *g* at 4°C for 20 min and milk samples for 10 min. Skim milk was collected while the fat was discarded. Skim milk was then centrifuged at 41,000 × *g* at 4°C for 45 min for colostrum and 20 min for milk. The resulting fraction was saved and frozen at -20°C for analysis of immunoglobulin and selenium content while the casein fraction was discarded.

### Whole blood selenium samples

Gilt and piglet whole blood Se concentrations in gilts and piglets samples were determined by fluorometric method [18] at the Olson Biochemistry Lab located on the campus of South Dakota State University.

### Statistical analysis

Effect of treatment on serum concentrations of immunoglobulins, whole blood concentrations of Se, and milk and colostral concentrations of immunoglobulins were analyzed by ANOVA for repeated measures in SAS by PROC MIXED [19]. The statistical model consisted of treatment, day, and their interactions. The effect of treatment was analyzed using litter within treatment as the error term, and the effects of day and any interaction were analyzed using the residual as the error term.

## Results

**Figure 1:**
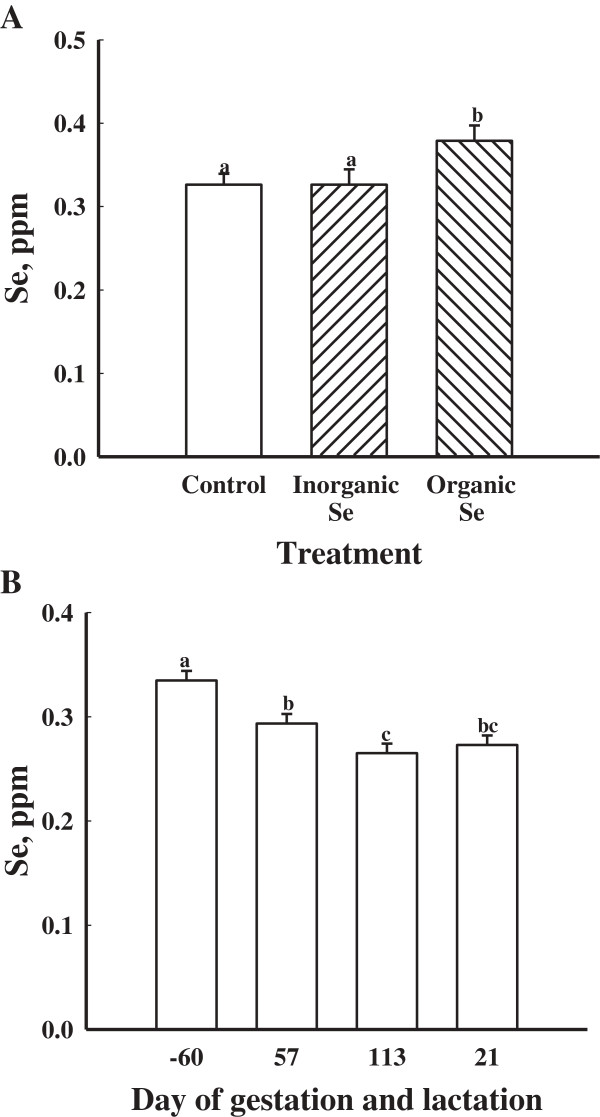
**Mean whole blood concentrations of Se in gilts fed no additional Se (Control; n = 8), inorganic Se (n = 4), and organic Se (n = 4), (A) and (B) mean whole blood concentrations of Se in gilts (n = 16) at day -60, 57, and 113 of gestation and d 21 of lactation.** Data are expressed as least-square means ± SEM. ^abc^Means with different letters differ (*P* < 0.05).

There were no treatment × day interactions (*P* > 0.05) for whole blood concentrations of Se or serum concentrations of IgG, IgA and IgM among the gilts supplemented with an organic, inorganic or no supplemental Se. Gilts supplemented with an organic source of Se had greater (*P* < 0.05) whole blood concentrations of Se than gilts supplemented with an inorganic source or no additional supplementation of Se (Figure 1A). Regardless of treatment, whole blood concentrations of Se were greatest (*P* < 0.05) at d -60 then decreased by d 57 of gestation reaching its lowest concentration by d 113 of gestation (*P* < 0.05; Figure 1B).

**Figure 2:**
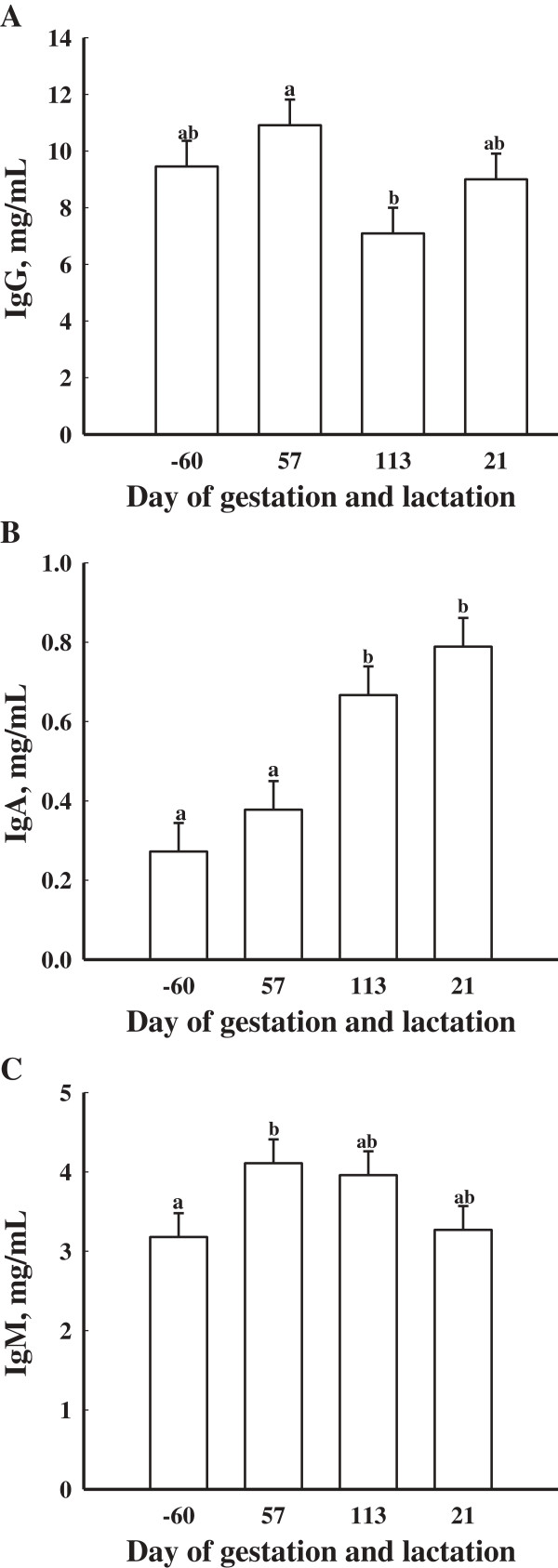
**Mean serum concentrations of IgG (A), IgA (B), and IgM (C) in gilts (n = 16) at d -60, 57, and 113 of gestation and d 21 of lactation.** Data are expressed as least-square means ± SEM. ^ab^Means with different letters differ (*P* < 0.05).

Serum concentrations of IgG did not differ (*P* > 0.05) in gilts fed inorganic and organic sources of Se (Table 2). However, there was an effect of time on serum concentrations of IgG. Gilts at d 57 of gestation had greater (*P* < 0.05) serum concentrations of IgG than gilts at d 113 of gestation (Figure 2A).

There were no differences due to dietary treatment on serum concentrations of IgA (*P* > 0.05; Table 2). There was an effect of day of gestation on serum concentrations of IgA as d 113 of gestation and d 21 of lactation were greater (*P* < 0.05) compared to d -60 and d 57 of gestation (Figure 2B). There was no treatment × day effect for serum concentrations of IgA (*P* > 0.05).

There was no effect of dietary treatment (*P* > 0.05) on serum concentrations of IgM (Table 2). There was a trend for serum concentrations of IgM being the greatest at d 57 of gestation compared to d -60 (*P* < 0.08; Figure 2C).

**Figure 3:**
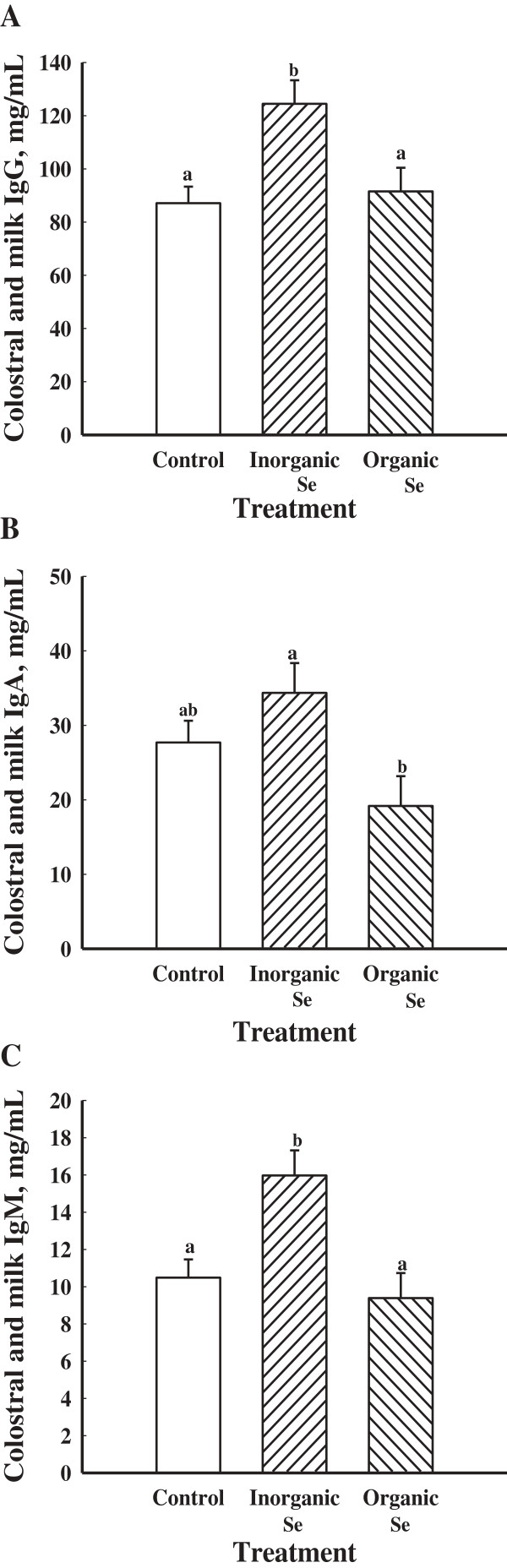
**Mean colostral and milk concentrations of IgG (A), IgA (B), and IgM (C) in gilts fed no additional Se (Control; n = 8), inorganic Se (n = 4), and organic Se (n = 4).** Data are expressed as least-square means ± SEM. ^ab^Means with different letters differ (*P* < 0.05).

**Figure 4:**
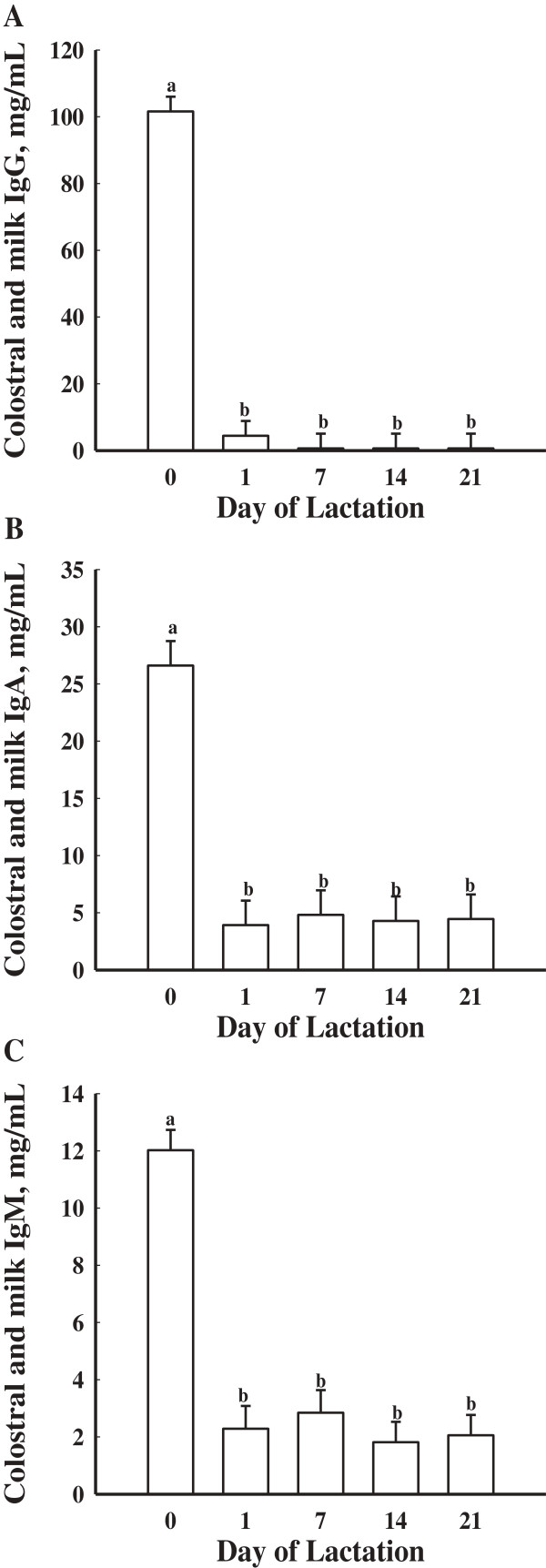
**Mean colostral and milk concentrations of IgG (A), IgA (B), and IgM (C) in gilts (n = 16) on d 0, 1, 7, 14 and 21 of lactation.** Day 0 represents the day of parturition. Data are expressed as least-square means ± SEM. ^ab^Means with different letters differ (*P* < 0.05).

There were no treatment × day interactions (*P* > 0.05) on colostral and milk concentrations of IgG, IgA and IgM among the gilts supplemented with an organic, inorganic or no supplemental Se. There was an overall effect of dietary treatment on colostral and milk concentrations of IgG (*P* < 0.05; Figure 3A). Gilts supplemented with an inorganic source of Se had greater (*P* < 0.05) colostral and milk concentrations of IgG compared to gilts supplemented with an organic source or no additional Se (Figure 3A). Colostral and milk concentrations of IgG were greatest at parturition (*P* < 0.05) then decreased by d 1 of lactation and remained low throughout the remainder of lactation (Figure 4A).

Gilts supplemented with an inorganic source of Se had greater (*P* < 0.05) colostral and milk concentrations of IgA than gilts supplemented with an organic source of Se (Figure 3B). Colostral and milk concentrations of IgA were greatest (*P* < 0.05) on the day of parturition (d 0) then decreased on d 1 and continued to be low throughout lactation (Figure 4B).

Colostral and milk concentrations of IgM were greatest (*P* < 0.05) in gilts supplemented with an inorganic source of Se compared to gilts supplemented with an organic source of Se or no additional Se (Figure 3C). Colostral and milk concentrations of IgM were greatest (*P* < 0.05) at parturition (d 0) then decreased throughout the remainder of lactation (Figure 4C).

**Figure 5:**
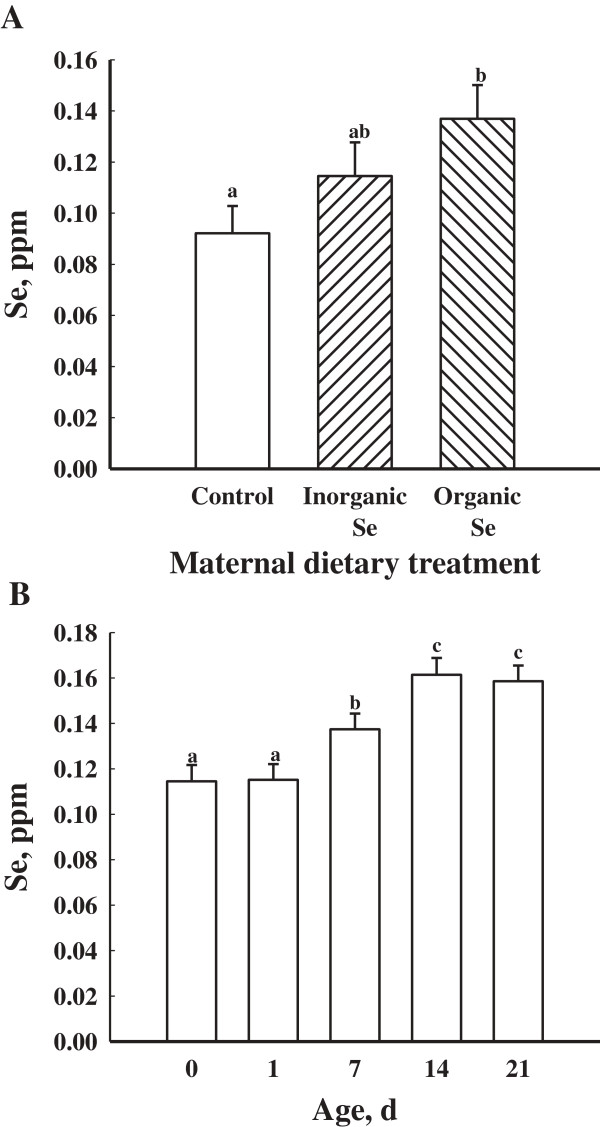
**Mean whole blood concentrations of Se in piglets from gilts supplemented with no additional Se (Control; n = 8 litters), gilts supplemented with inorganic Se (n = 4 litters), and gilts supplemented with organic Se (n = 4 litters) (A) and mean whole blood concentrations of Se in piglets (n = 16 litters) at d 0, 1, 7, 14 and 21 of age (B).** Day 0 represents the day of parturition. Data are expressed as least-square means ± SEM. ^abc^Means with different letters differ (*P* < 0.05).

Maternal dietary treatment affected (*P* < 0.05) the whole blood concentrations of Se in piglets (Figure 5A). Piglets born to gilts supplemented with an organic source of Se had greater (*P* < 0.05) whole blood concentrations of Se than piglets from gilts supplemented with no additional Se (Figure 5A). There was no effect of maternal dietary treatment on litter size (*P* > 0.05). Piglet whole blood concentrations of Se were greatest (*P* < 0.05) at d 14 and 21 of age compared to d 0, 1, and 7 (Figure 5B). Additionally, seven-day-old piglets had greater (*P* < 0.05) whole blood concentrations of Se compared to d 1 and d 0 piglets (Figure 5B).

**Figure 6:**
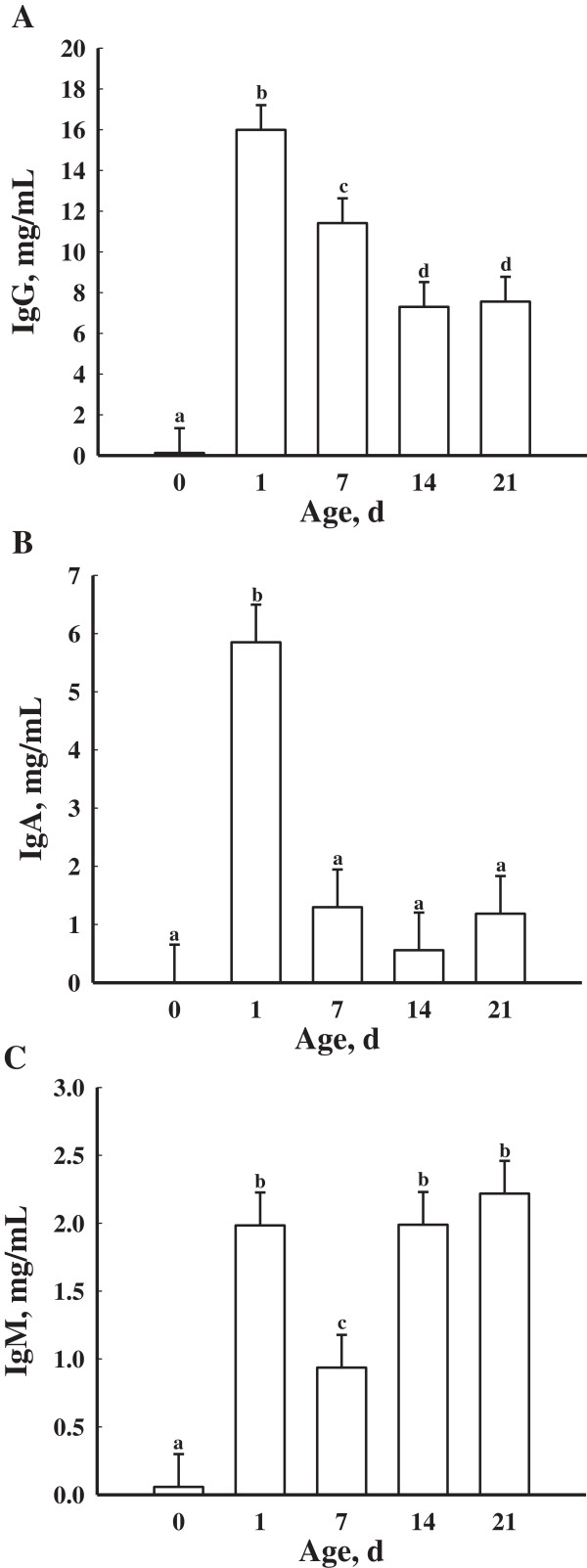
**Mean serum concentrations of IgG (A), IgA (B), and IgM (C) in piglets (n = 16 litters) at d 0, 1, 7, 14 and 21 of age.** Day 0 represents the day of parturition. Data are expressed as least-square means ± SEM. ^abcd^Means with different letters differ (*P* < 0.05).

No treatment or treatment × day effect was found for serum concentrations of IgG in piglets (*P* > 0.05). Serum concentrations of IgG in piglets differed by day (*P* < 0.05; Figure 6A). Serum concentrations of IgG in piglets on d 0 were decreased (*P* < 0.05) compared to all other days. Serum concentrations of IgG in piglets were greatest (*P* < 0.05) on d 1 compared to all other days. Seven day old piglets had greater (*P* < 0.05) serum concentrations of IgG than d 14 and 21 piglets.

Serum concentrations of IgA in piglets did differ by day (*P* < 0.05; Figure 6B) but not by treatment (*P* > 0.05). Serum concentrations of IgA were greatest (*P* < 0.05) on d 1 compared to all other days. Additionally, there was no treatment × day interaction (*P* > 0.05).

Serum concentrations of IgM were not affected by treatment and no day × treatment effect was found (*P* > 0.05). Serum concentrations of IgM in piglets did differ by day (*P* < 0.05; Figure 6C). Serum concentrations of IgM in piglets on d 0 were decreased (*P* < 0.05) compared to all other days. Serum concentrations of IgM in piglets on d 1, 14, and 21 were greater (*P* < 0.05) than on d 0 and 7.

## Discussion

The Se concentrations in the diets of the current study are greater than those reported in other studies [4,5,7]. In the current study, it was the intention to create maternal diets which were reflective of diets typically fed to gestating and lactating swine. Most pigs raised in the midwestern United States consume grains that are grown on soils which have moderate to high Se concentrations; therefore, the grains in this region are greater in Se compared to most other parts of the U.S. Also, the swine industry routinely adds Se to diets at 0.3 ppm as dictated by the FDA, regardless of the endogenous Se content of the grain.

In the current study, whole blood concentrations of Se were greater in gilts that were supplemented with organic Se. This is similar to the findings of Yoon and McMillian [7] who reported greater whole blood concentrations of Se at farrowing when females were fed organic Se. However, Mahan and Kim [4] observed no differences in blood concentrations of Se from females fed either organic or inorganic Se at 0.3 ppm. Whole blood concentrations of Se for gilts also decreased from the start of the trial to parturition regardless of the treatments in the current study. These results also correspond with studies done by Mahan and Peters [7] and Yoon and McMillan [7] as they all documented a decrease in whole blood concentrations of Se from the initial start of the trial at gestation to parturition. Mahan and Kim [4] hypothesized the decline in Se concentration from gestation to parturition could demonstrate a need for the female to transfer Se to developing fetuses or for Se to produce selenoproteins that could then be transferred to either fetal or mammary tissue.

The greater whole blood concentration of Se in the gilts from this study compared to those reported by Kim and Mahan [20] can be attributed to the greater dietary concentration of Se in the diet. When comparing the results of the current study to those of Kim and Mahan [20], both studies fed diets supplemented with 0.3 ppm. The current study reported gilt whole blood Se levels of 0.33 to 0.28 ppm throughout the course of the trial, which was greater than those reported by Kim and Mahan [20] and Yoon and McMillan [7]. While previous studies [7,20] supplemented Se at 0.3 ppm as inorganic and organic Se, the discrepancy among studies for increased concentrations of Se may be attributed to gilts being fed their respective diets for 60 d before breeding as Yoon and McMillan [7] did not start feeding the treatment diets until 60 d prepartum.

Whole blood concentrations of Se were greatest for piglets born to gilts that received diets supplemented with organic Se in the current study. This is in agreement with the observations of Mahan and Kim [4] and Kim and Mahan [20] who reported greater piglet whole blood concentrations of Se from dams that were supplemented with organic Se. Though we did not have a day by treatment interaction effect, the whole blood concentrations of Se in piglets increased from d 1 of birth until weaning at d 21. Whole blood concentrations of Se were greatest on d 14 in piglets born to females supplemented with organic Se [5]. Yoon and McMillan [7] reported that piglets born to females receiving diets supplemented with organic Se had greater whole blood concentrations of Se than piglets born to females fed the control diet. The Yoon and McMillan [7] results are similar to the data reported in the current study as piglets from gilts who received diets supplemented with organic Se had greater whole blood concentrations of Se than piglets from gilts fed the control diet, but were not different than the piglets from gilts supplemented with inorganic Se. The current study did find greater values of Se in piglets, but it could be attributed to the maternal treatment diets being fed throughout the course of the pregnancy.

Altered thyroid hormone status may provide an explanation for the mechanism leading to an altered IgG absorption in dams that were supplemented with Se throughout gestation [21]. Fetal infusion of thyroid hormones resulted in decreased IgG absorption in lambs [22]. Selenium supplementation directly to calf colostrum increased serum concentrations of IgG in calves, but as the supplementation of Se increased the IgG absorption was impaired [11]. Kamada et al. [11] speculated that increased IgG absorption resulting from supplementation with Se was pharmacological and not nutritional. In the present study, the addition of either type of Se source had no effect on any of the serum immunoglobulins evaluated. Sows that were administered a Se injection on d 100 of gestation had decreased levels of serum immunoglobulins [16] compared to the serum immunoglobulins concentrations in the current study. The decreased serum concentrations of immunoglobulins could be attributed to only a single injection of Se or that the females used in the study had a more developed immune system [16].

Serum concentrations of IgG in piglets in the current study were greater than those reported by Yoon and McMillan [7]. Additionally, maternal dietary treatment had no effect on serum concentrations of IgG in piglets, which was also observed by Yoon and McMillan [7]. Increasing the concentration of circulating immunoglobulins in the neonatal pig is beneficial for long term piglet survival after weaning when the dam’s milk is no longer available. Immunoglobulin G is the predominant immunoglobulin in colostrum and has a half-life of 14 d, while IgA and IgM have half-lives of 2.5 and 5 d, respectively [23]. A decrease in serum concentrations of IgG were observed over time. Serum concentrations of IgG were greatest on d 1 because of the high ingestion of colostral IgG from the gilts. Serum concentrations of IgM were greater on d 1 and then decreased by d 7 indicating that the IgM transferred from the colostrum had decreased in concentration, but concentrations of IgM were increased by d 14 and 21. This increase in concentrations of IgM may indicate that the piglet immune system is maturing.

The limited numbers of animals in the experimental groups in the present study probably precluded us from finding differences in many of the measured parameters. However, colostral and milk concentrations of immunoglobulins were increased in the sows treated with inorganic Se which could further protect the neonatal pig. When ewes were supplemented with supranutritional levels of inorganic or organic Se, colostral IgG concentrations increased but it was dependent upon the dosage of Se administered [24]. Additionally, Se supplementation may affect cytokine production as has been observed in the horse [25]. Horses receiving an inorganic source of Se had the greatest mean expression of IL-8 by stimulated neutrophils. Interleukin-8 can induce chemotaxis which is important for the recruitment of leukocytes to the site of infection as well as activation of neutrophils [26]. Thus, based upon our observations and the corollary work by others, further research examining cytokine profiles and/or dosage and source of Se administered to the pig is warranted.

Early weaning of piglets can reduce performance, which correlates to increased susceptibility to disease [27]. Piglets weaned at an older age may be more efficient at mounting an immune response because of their more developed immune system [28]. As weaning age increased, a reduction in plasma IgG concentration was observed and this reduction could reflect the stage of development of the innate immune system [28]. Swine producers are always examining ways to decrease piglet mortality. Weaning at an age when piglets are finally developing their own immune system can decrease mortality in finishing pigs Early weaning piglets to an isolated site can reduce the potential for disease transfer from the dam [29] and decrease immunological stress [30]. Early weaning to an isolated site improved growth and feed efficiency compared to piglets weaned to a conventional farrow-to-finish system [31]. Early weaning practices should be made to a clean environment as Blecha et al. [32] showed that early weaning compromises the cellular immunity of these younger animals. The practice of early weaning is stressful to the piglet as their immune system is not fully developed and considerations should be made as to what age the piglets weaning occurs.

## Conclusion

The results of this study are similar to previously published reports, but the current study also demonstrates the effect of Se sources on passive transfer of immunoglobulins. The addition of inorganic or organic sources of Se in the maternal diet did not affect immunoglobulin concentration in the pig, but we were able to profile the concentrations of immunoglobulins over a 3 wk period in the piglet, which may help in determining when the piglet immune system is starting to mature.

## Competing interests

None of the authors have any competing interest to declare.

## Authors’ contributions

All authors have made substantial contributions to: the research design, analysis or interpretation of data, and in drafting the paper. All authors read and approved the final manuscript.
